# Author Correction: Defective DNA damage repair leads to frequent catastrophic genomic events in murine and human tumors

**DOI:** 10.1038/s41467-026-71976-x

**Published:** 2026-04-20

**Authors:** Manasi Ratnaparkhe, John K. L. Wong, Pei-Chi Wei, Mario Hlevnjak, Thorsten Kolb, Milena Simovic, Daniel Haag, Yashna Paul, Frauke Devens, Paul Northcott, David T. W. Jones, Marcel Kool, Anna Jauch, Agata Pastorczak, Wojciech Mlynarski, Andrey Korshunov, Rajiv Kumar, Susanna M. Downing, Stefan M. Pfister, Marc Zapatka, Peter J. McKinnon, Frederick W. Alt, Peter Lichter, Aurélie Ernst

**Affiliations:** 1 Division of Molecular Genetics, German Cancer Consortium (DKTK), German Cancer Research Center (DKFZ); Faculty of Biosciences, Heidelberg University Germany, Heidelberg, 69120 Germany; 2 Division of Molecular Genetics, German Cancer Consortium (DKTK), German Cancer Research Center (DKFZ), Heidelberg, 69120 Germany; 3 Boston Children’s Hospital, Howard Hughes Medical Institute and Department of Genetics, Harvard Medical School, Boston, 02115 MA USA; 4 Hopp Children’s Cancer Center at the NCT Heidelberg (KiTZ), Heidelberg, 69120 Germany; 5 Division of Pediatric Neurooncology, German Cancer Research Center (DKFZ), Heidelberg, 69120 Germany; 6 Department of Developmental Neurobiology, St. Jude Children’s Research Hospital, Memphis, TN 38105-3678 US; 7 Institute of Human Genetics, University of Heidelberg, Heidelberg, 69120 Germany; 8 Department of Pediatrics, Oncology, Hematology and Diabetology, Medical University of Lodz, Lodz, 91-738 Poland; 9 Clinical Cooperation Unit Neuropathology, German Cancer Research Center (DKFZ), Department of Neuropathology, Heidelberg University Hospital and German Cancer Consortium (DKTK), Heidelberg, 69120 Germany; 10 Division of Molecular Genetic Epidemiology; German Consortium for Translational Cancer Research (DKTK), German Cancer Research Center, Heidelberg, 69120 Germany; 11 Department of Genetics, St. Jude Children’s Research Hospital, Memphis, 38105-3678 TN USA

Correction to: *Nature Communications* 10.1038/s41467-018-06925-4, published online 12 November 2018

In the version of this article initially published, due to a figure preparation mistake, in Fig. 5a, bottom row of NSC *gTP53 gLIG4*, second-to-last image on right, the image presented was incorrect. Due to the age of the article, the figure cannot be updated directly. The correct image is available as Supplementary Information accompanying this amendment. This notice serves to correct the article.

## Supplementary information

Corrected Fig. 5

