# Supplementary material for: Defective DNA damage repair leads to frequent catastrophic genomic events in murine and human tumors
Source: Nat Commun. 2018 Nov 12;9:4760. doi: 10.1038/s41467-018-06925-4 (PMC6232171; doi:10.1038/s41467-018-06925-4)
Supplement: Supplementary file 1 — Supplementary Information [file 41467_2018_6925_MOESM1_ESM.pdf]

Supplementary Information

Defective DNA damage repair leads to frequent catastrophic genomic events in murine and human tumors

Ratnaparkhe,Wong *et. al.*

a.

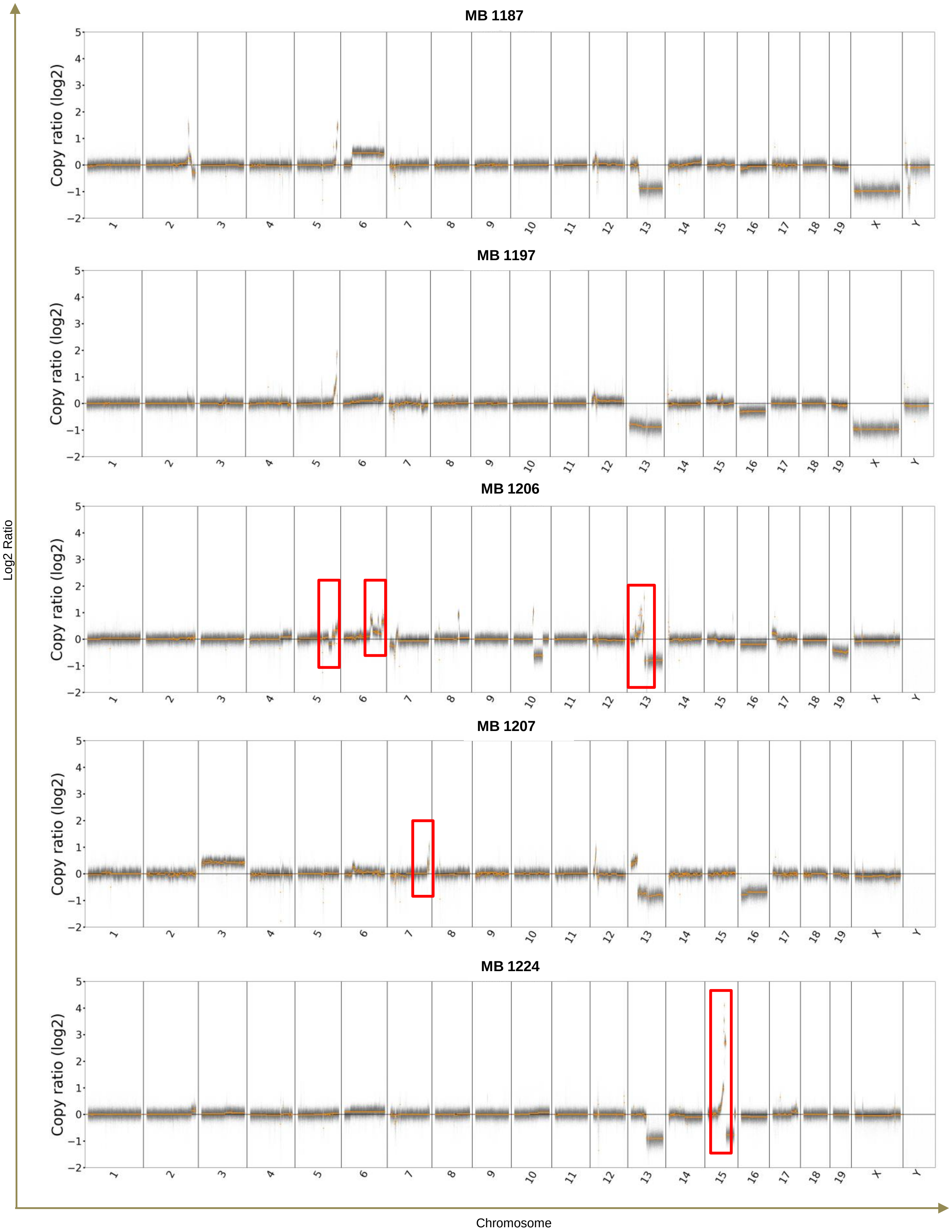

MB 1187
MB 1197
MB 1206
MB 1207
MB 1224
Copy ratio (log2)
Log2 Ratio
Chromosome
1
2
3
4
5
6
7
8
9
10
11
12
13
14
15
16
17
18
19
X
Y

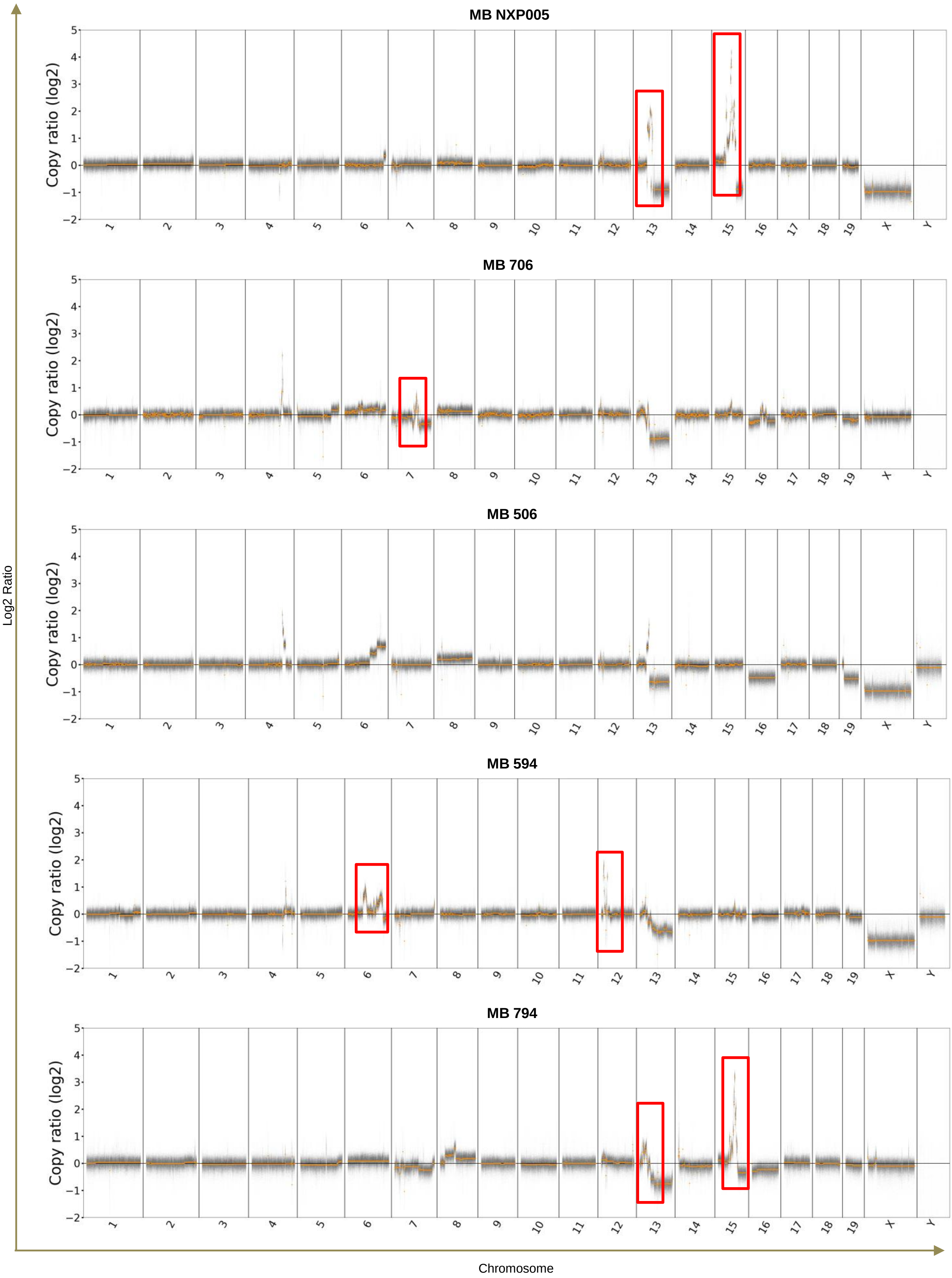
MB NXP005
MB 706
MB 506
MB 594
MB 794
Copy ratio (log2)
Log2 Ratio
Chromosome
1
2
3
4
5
6
7
8
9
10
11
12
13
14
15
16
17
18
19
X
Y

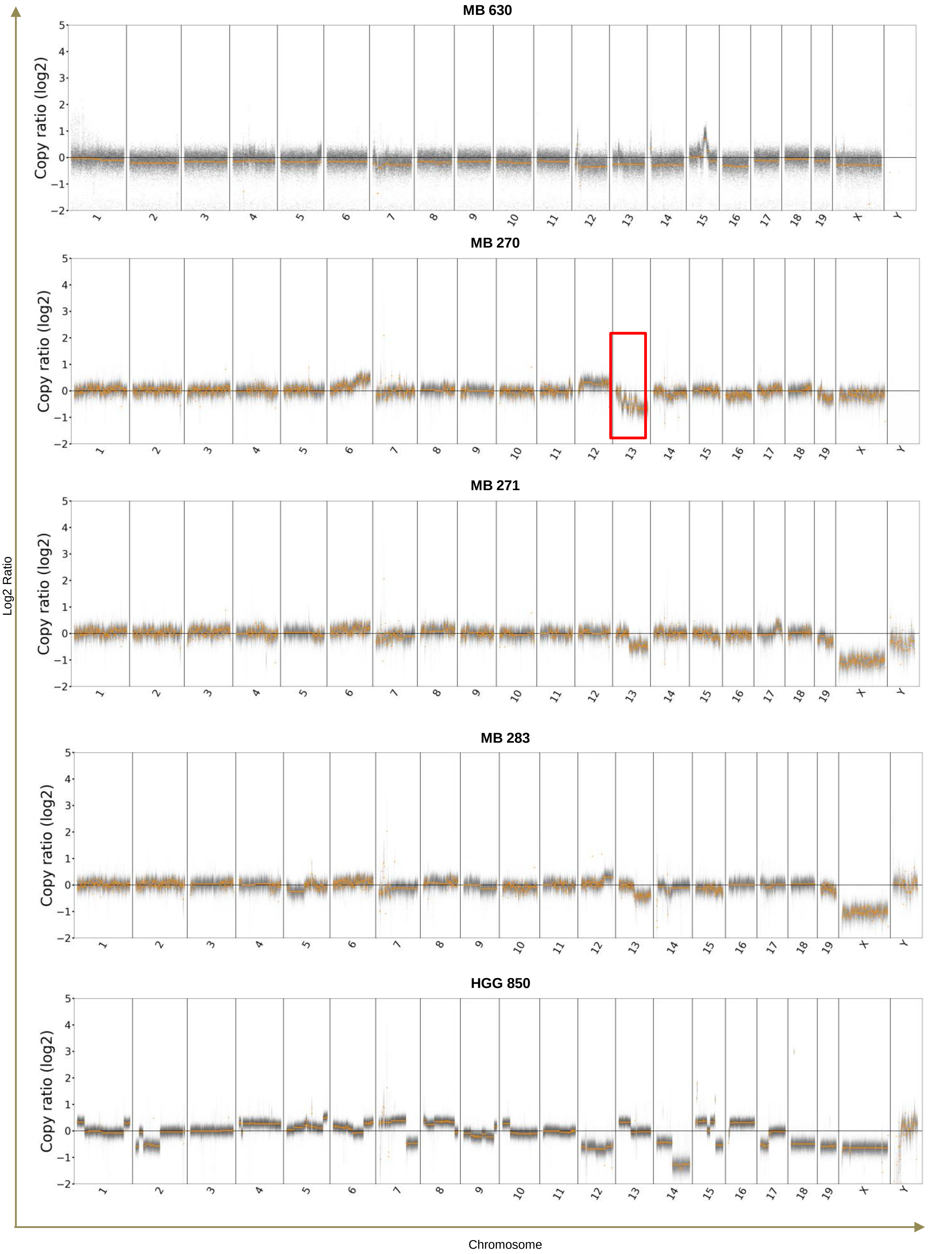
MB 630
MB 270
MB 271
MB 283
HGG 850
Copy ratio (log2)
Log2 Ratio
Chromosome
1
2
3
4
5
6
7
8
9
10
11
12
13
14
15
16
17
18
19
X
Y

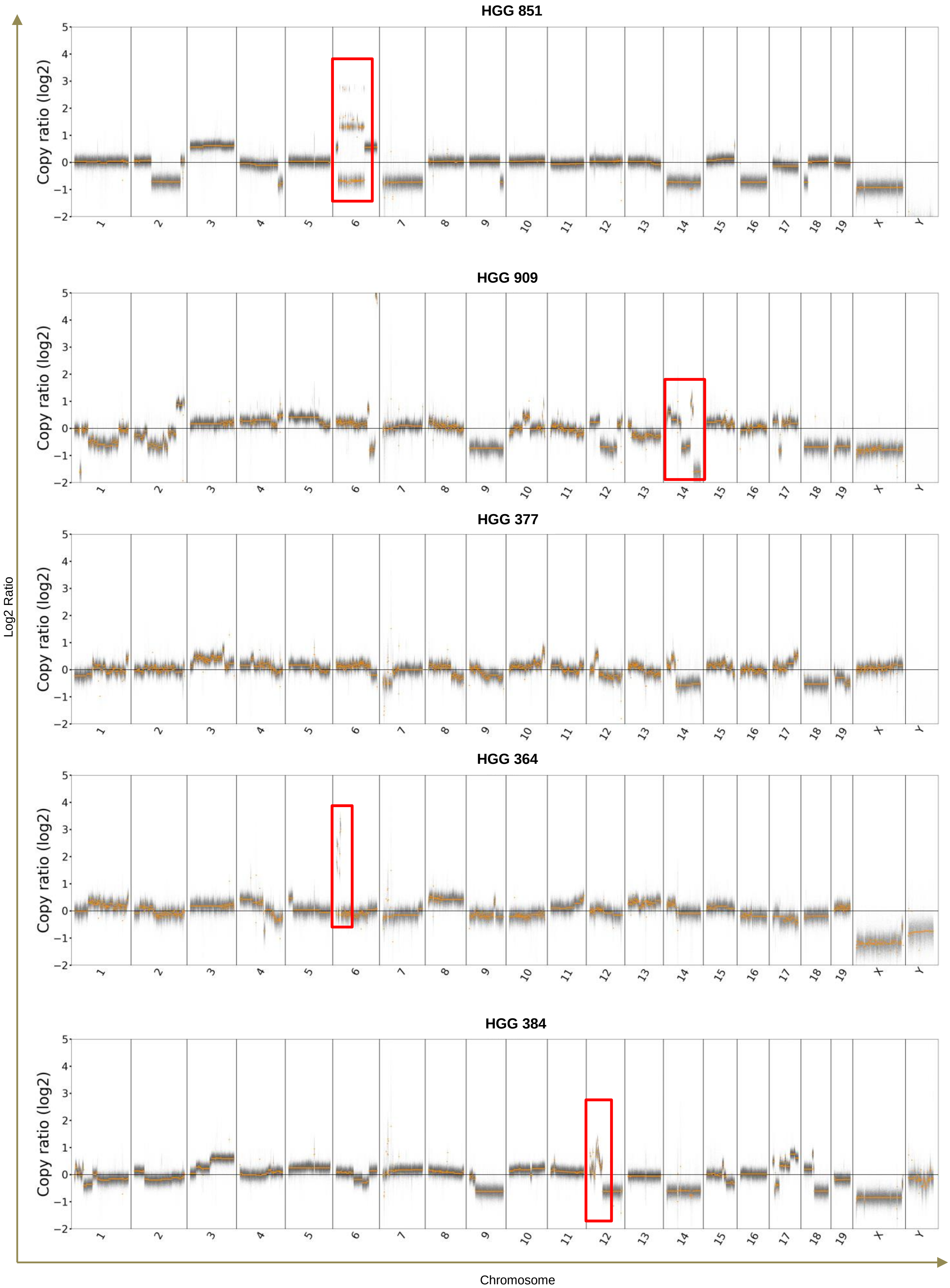
HGG 851
HGG 909
HGG 377
HGG 364
HGG 384
Copy ratio (log2)
Log2 Ratio
Chromosome
1
2
3
4
5
6
7
8
9
10
11
12
13
14
15
16
17
18
19
X
Y

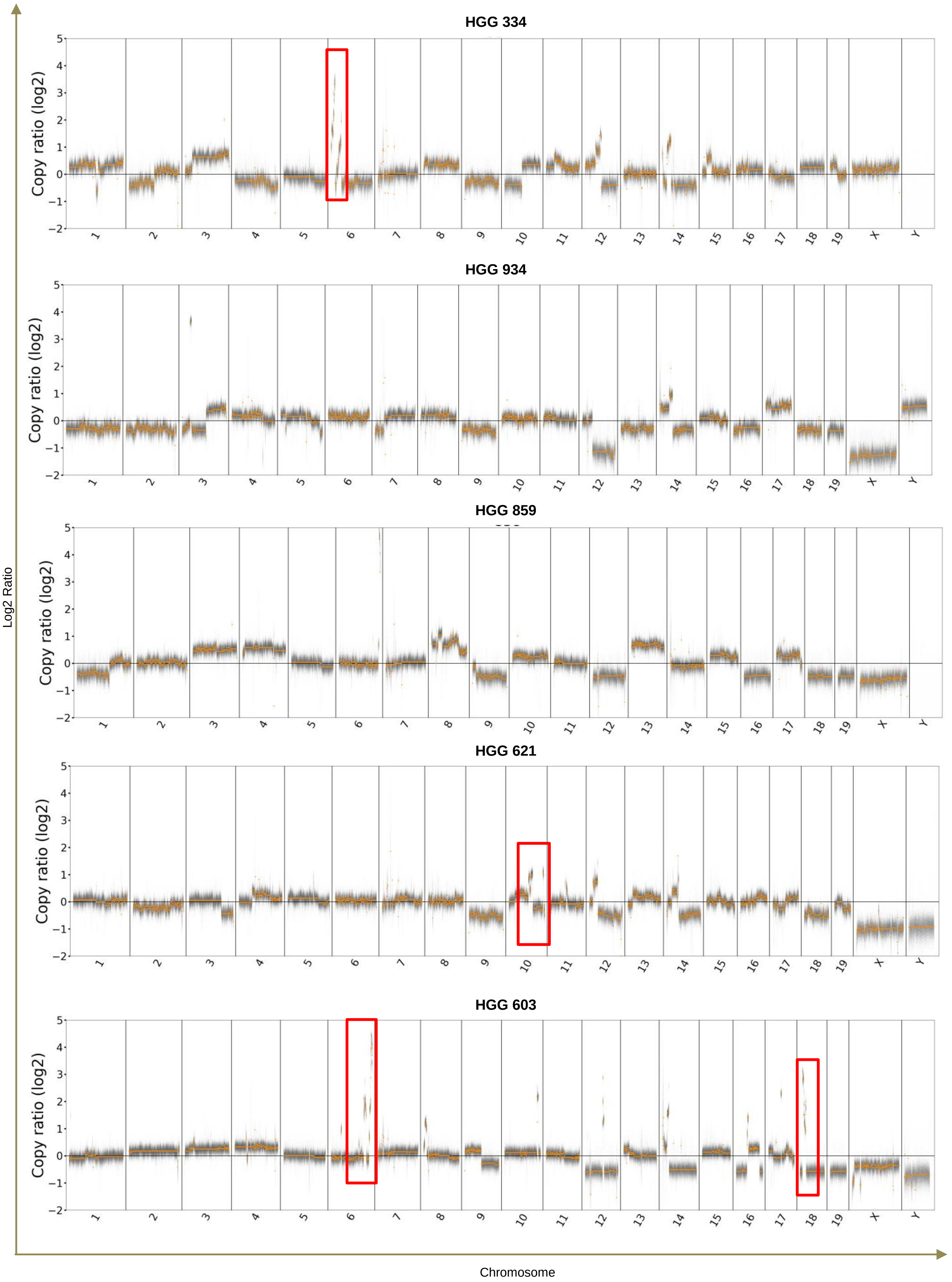

HGG 334
HGG 934
HGG 859
HGG 621
HGG 603
Copy ratio (log2)
1
2
3
4
5
6
7
8
9
10
11
12
13
14
15
16
17
18
19
X
Y
Log2 Ratio
Chromosome

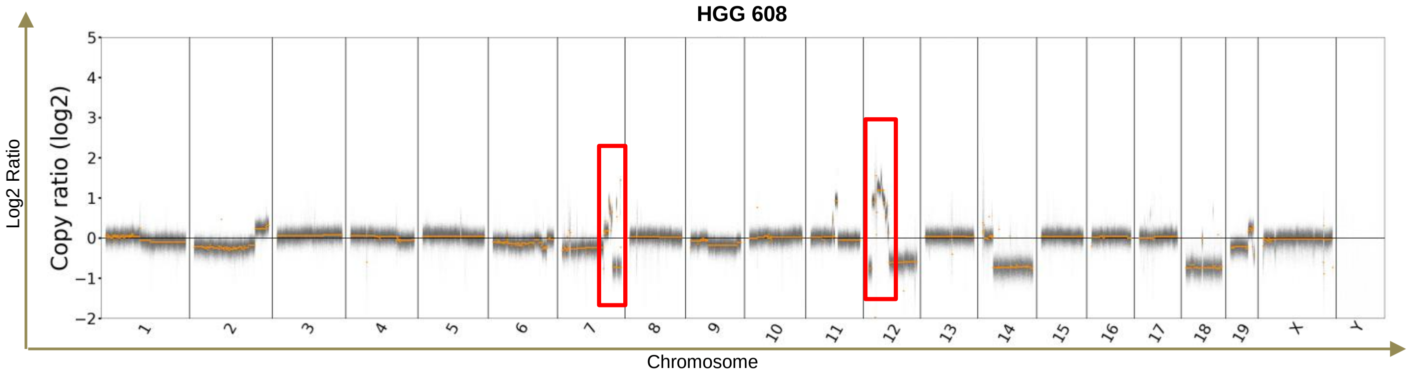
HGG 608
Log2 Ratio
Copy ratio (log2)
5
4
3
2
1
0
−1
−2
1 2 3 4 5 6 7 8 9 10 11 12 13 14 15 16 17 18 19 X Y
Chromosome

b.

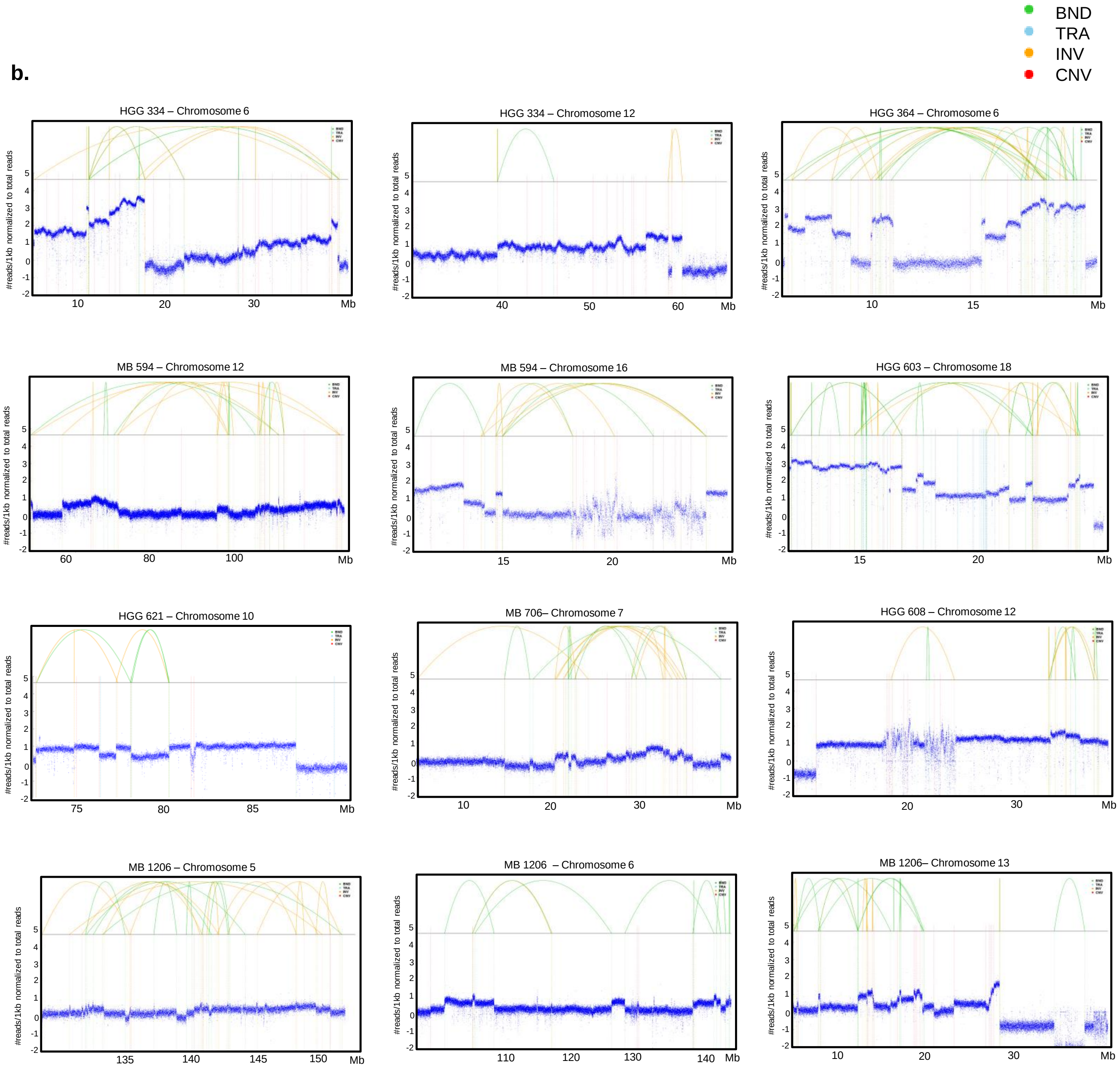
BND
TRA
INV
CNV
HGG 334 – Chromosome 6
HGG 334 – Chromosome 12
HGG 364 – Chromosome 6
MB 594 – Chromosome 12
MB 594 – Chromosome 16
HGG 603 – Chromosome 18
HGG 621 – Chromosome 10
MB 706– Chromosome 7
HGG 608 – Chromosome 12
MB 1206 – Chromosome 5
MB 1206 – Chromosome 6
MB 1206– Chromosome 13
#reads/1kb normalized to total reads
Mb

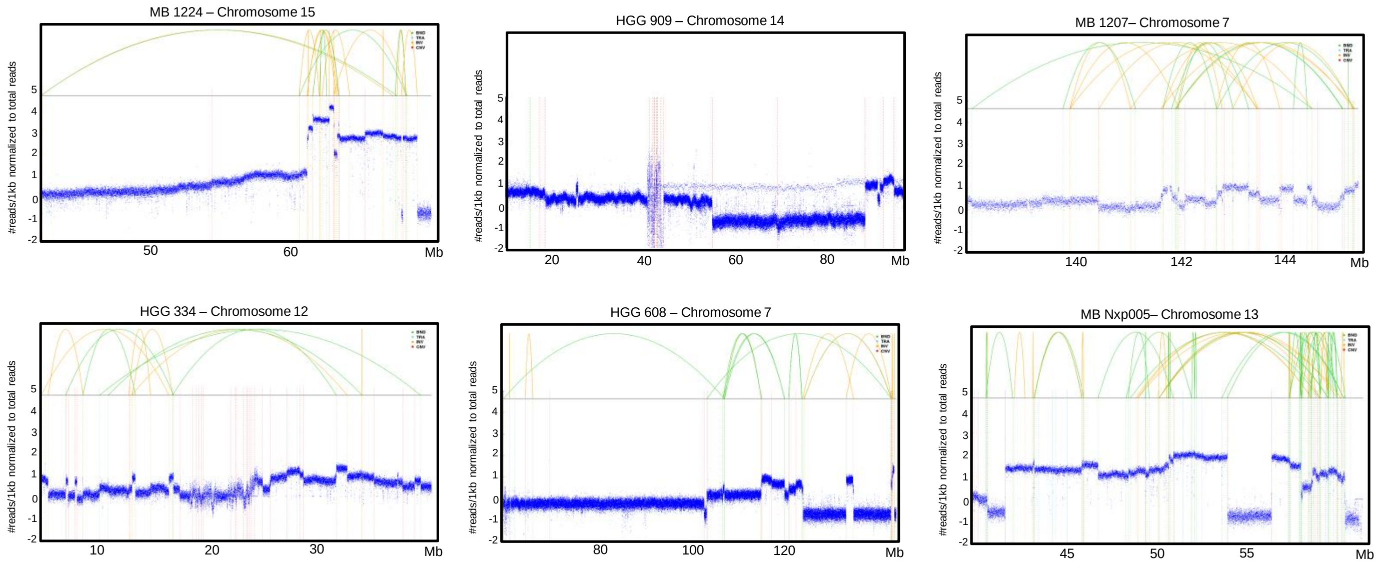
MB 1224 – Chromosome 15
HGG 909 – Chromosome 14
MB 1207– Chromosome 7
HGG 334 – Chromosome 12
HGG 608 – Chromosome 7
MB Nxp005– Chromosome 13
#reads/1kb normalized to total reads
Mb
BND
TRA
INV
CNV

c.

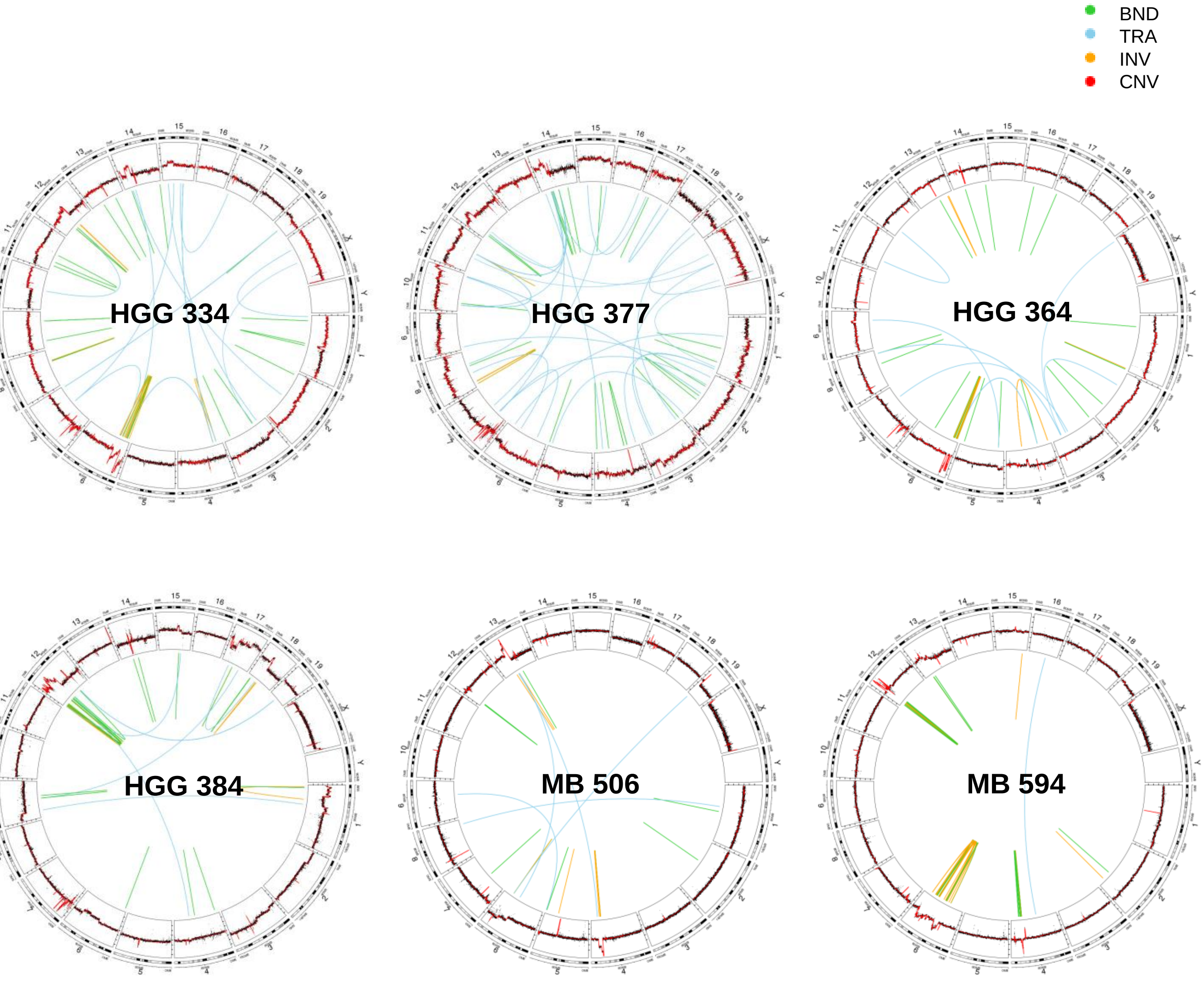
BND
TRA
INV
CNV
HGG 334
HGG 377
HGG 364
HGG 384
MB 506
MB 594

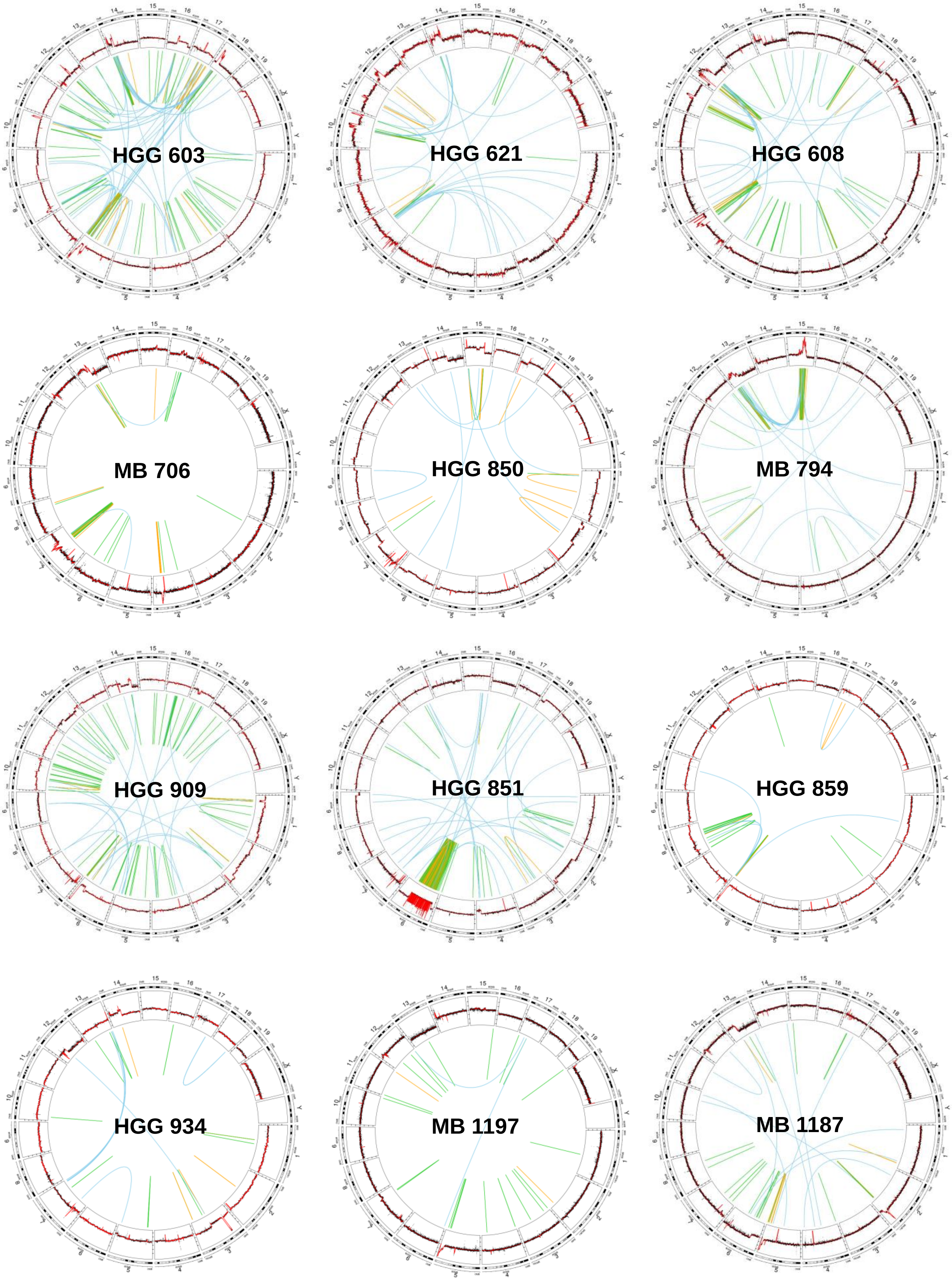
HGG 603
HGG 621
HGG 608
MB 706
HGG 850
MB 794
HGG 909
HGG 851
HGG 859
HGG 934
MB 1197
MB 1187

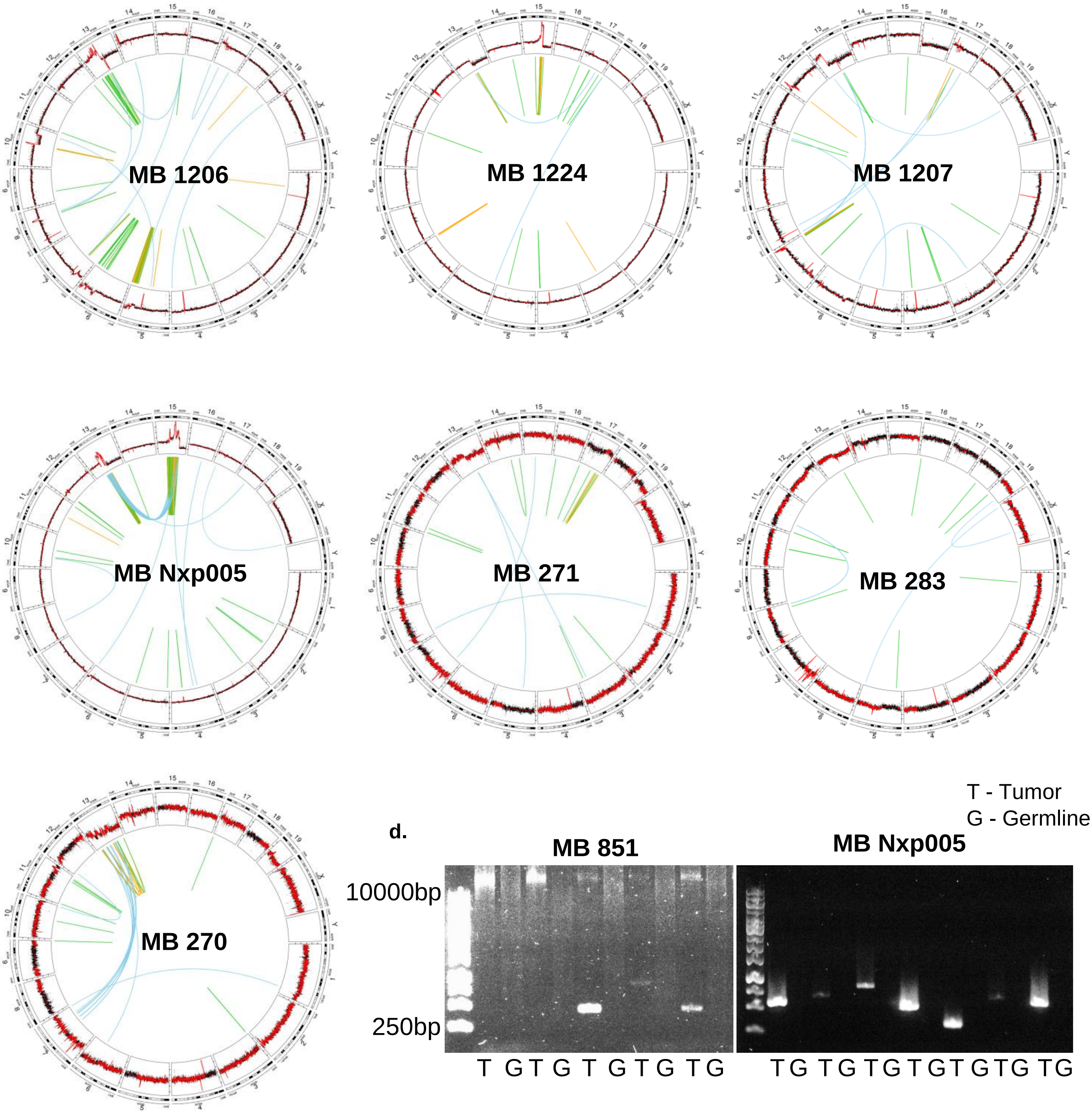

Supplementary Figure 1 : **Coverage plots for all analyzed murine tumors, CIRCOS plots and blow-out views of the chromosomes affected by complex rearrangements and validation of a subset of structural variants by PCR**.

a. Coverage plots for all analyzed murine tumors. Red boxes show chromosomes affected by complex genome rearrangements. Blow-out views of all affected regions are shown in b and circos plots in c. **b.** Blow-out views for regions affected by complex genome rearrangements in all murine tumors. BND, break end; TRA, translocation; INV, inversion; CNV, copy-number variation. **c.** CIRCOS plots for all murine tumors. BND, break end; TRA, translocation; INV, inversion; CNV, copy-number variation. **d.** Validation of a subset of rearrangements by PCR. For 12 of the 14 breakpoints for which we designed primers, we obtained a PCR product at the expected size.

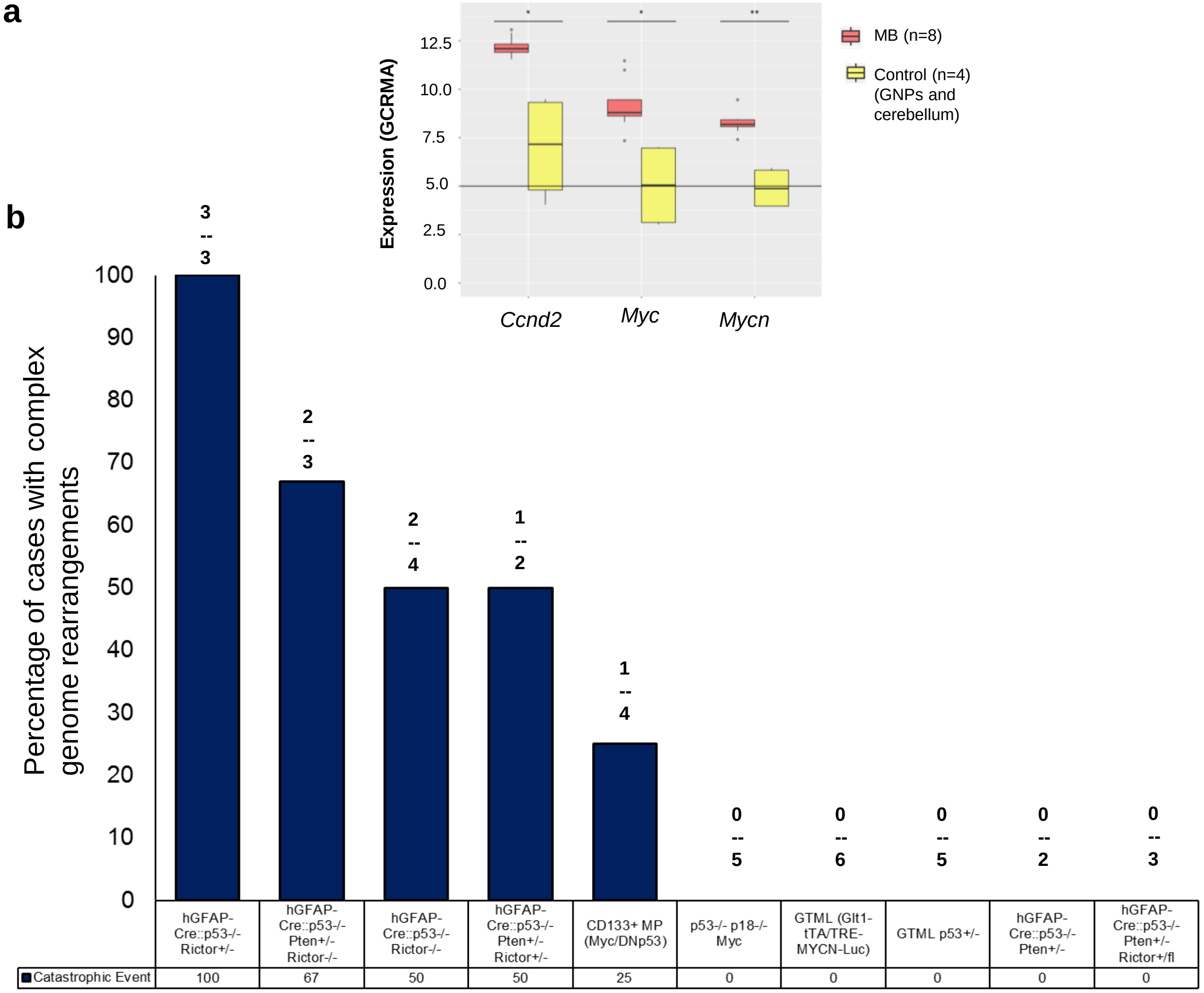

MycN Driven	No*	No*	No*	No*	No	No	Yes	Yes	No	No
cMyc Driven	No	No	No	No	Yes	Yes	No	No	No	No
Tp53 Status	p53-/-	p53-/-	p53-/-	p53-/-	p53DN	p53-/-	p53+/+	p53+/-	p53-/-	p53-/-
Tumor/Subgroup	MB/SHH	MB/SHH	MB/SHH	MB/SHH	MB/SHH	MB/SHH	MB/Group3	MB/Group4	MB/SHH	MB/SHH

*Note: these models show *MycN* amplification as a second hit in 33% of the analyzed tumors
p53DN, p53 Dominant Negative; MB, Medulloblastoma; SHH, Sonic Hedgehog

Supplementary Figure 2: Complex genome rearrangements drive tumor development and frequency of complex rearrangements in DNA repair proficient murine MBs and in MYC/MYCN-driven MB models with various p53 status

a. Complex genome rearrangements drive tumor development. Driver genes located in the chromosome regions affected by the catastrophic events are overexpressed. Expression levels for selected genes from Affymetrix expression arrays for medulloblastomas from XRCC4/p53 deficient mice (MB, n=8) and normal brain from animals of the same genotype (Control, n=4; 2 from whole cerebella and 2 from isolated cerebellar granule neural progenitors, shown together on the yellow box plots). Unpaired t-tests were used to test for significance. **b.** Frequency of complex genome rearrangements in DNA repair proficient mouse models of medulloblastoma of various p53 status. Re-analysis of low-coverage WGSeq data from published studies1, 2. From the 10 mouse models (3 to 6 mice per genotype), 4 are MYC- or MYCN-driven (see table below). A subset of the MBs from the mouse models not based on Myc/Mycn overexpression show Myc/Mycn amplifications (marked with asterisks).

a.

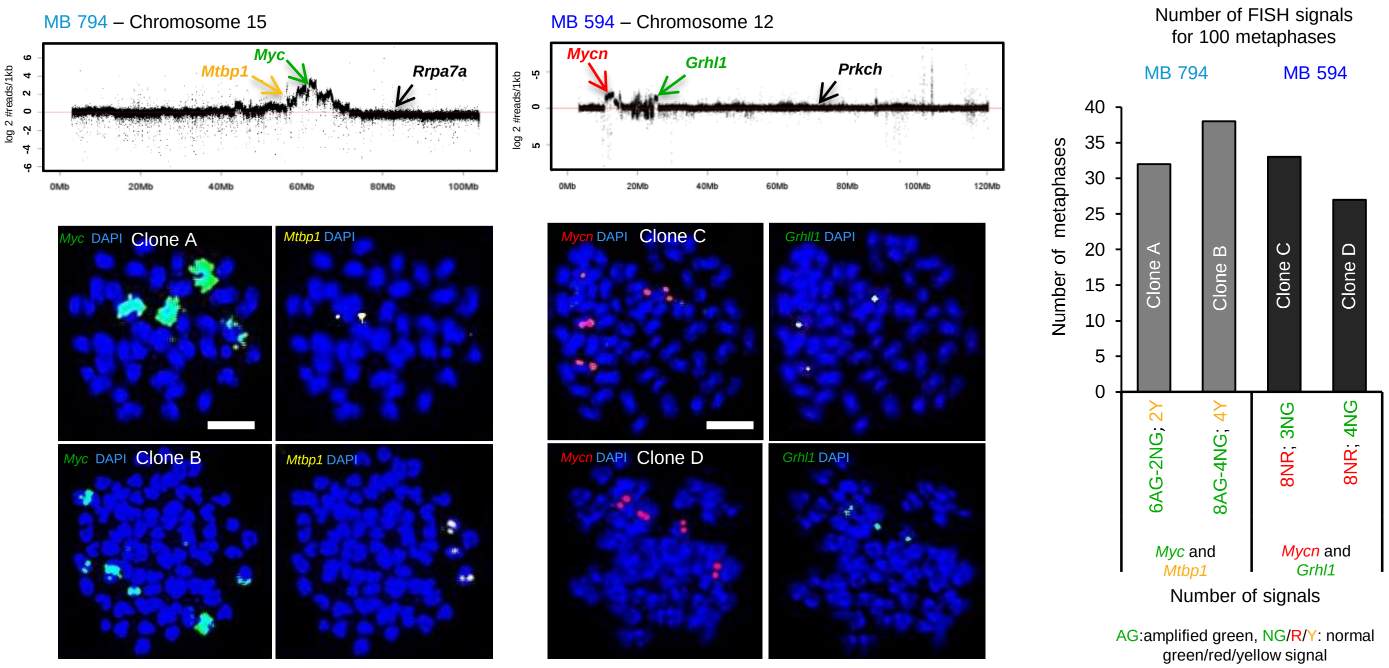
MB 794 – Chromosome 15
Mtbp1
Myc
Rrpa7a
log 2 #reads/1kb
0Mb 20Mb 40Mb 60Mb 80Mb 100Mb
MB 594 – Chromosome 12
Mycn
Grhl1
Prkch
0Mb 20Mb 40Mb 60Mb 80Mb 100Mb 120Mb
Myc DAPI Clone A
Mtbp1 DAPI
Myc DAPI Clone B
Mtbp1 DAPI
Mycn DAPI Clone C
Grhl1 DAPI
Mycn DAPI Clone D
Grhl1 DAPI
Number of FISH signals
for 100 metaphases
MB 794
MB 594
Number of metaphases
40 35 30 25 20 15 10 5 0
Clone A
Clone B
Clone C
Clone D
6AG-2NG; 2Y
8AG-4NG; 4Y
8NR; 3NG
8NR; 4NG
Myc and
Mtbp1
Mycn and
Grhl1
Number of signals
AG:amplified green, NG/R/Y: normal
green/red/yellow signal

b

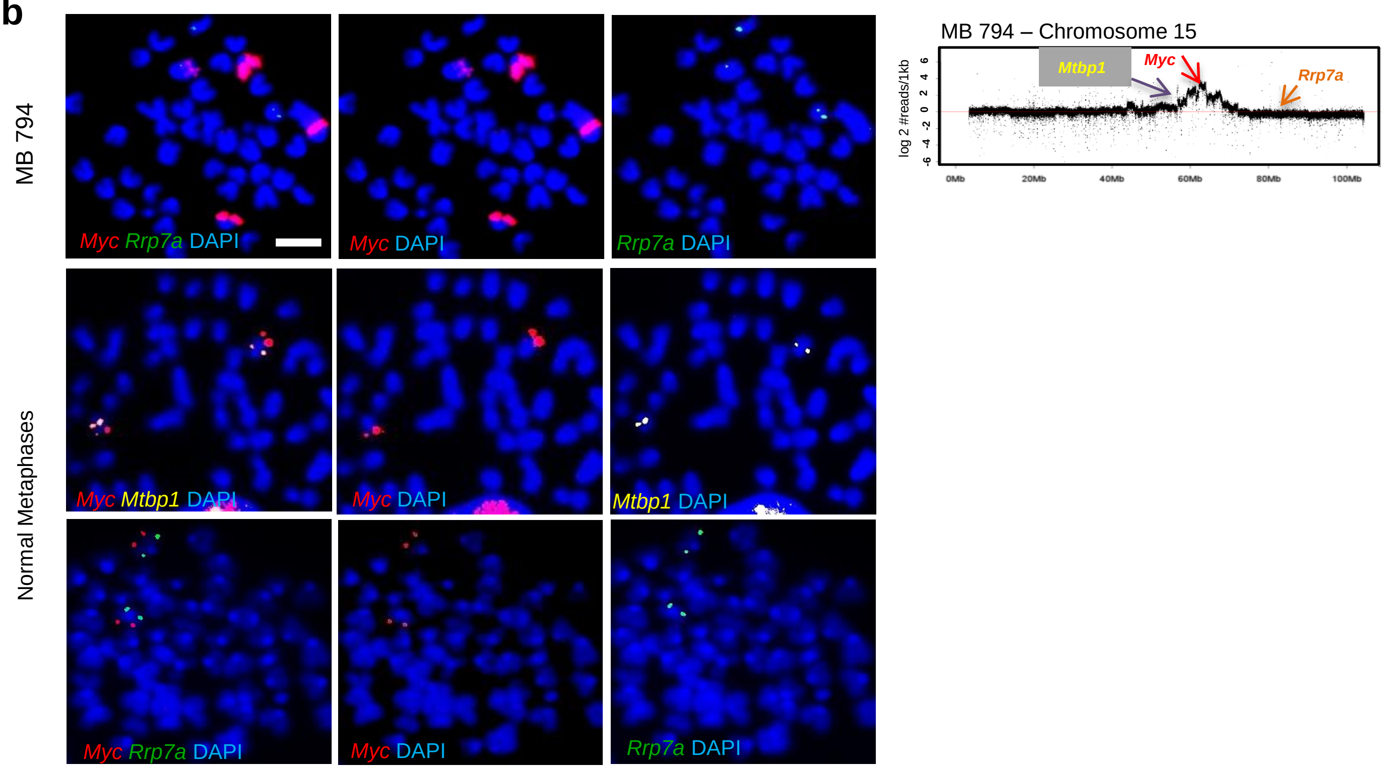
MB 794
Myc Rrp7a DAPI
Myc DAPI
Rrp7a DAPI
Normal Metaphases
Myc Mtbp1 DAPI
Myc DAPI
Mtbp1 DAPI
Myc Rrp7a DAPI
Myc DAPI
Rrp7a DAPI
MB 794 – Chromosome 15
Mtbp1
Myc
Rrp7a
log 2 #reads/1kb
0Mb 20Mb 40Mb 60Mb 80Mb 100Mb

c

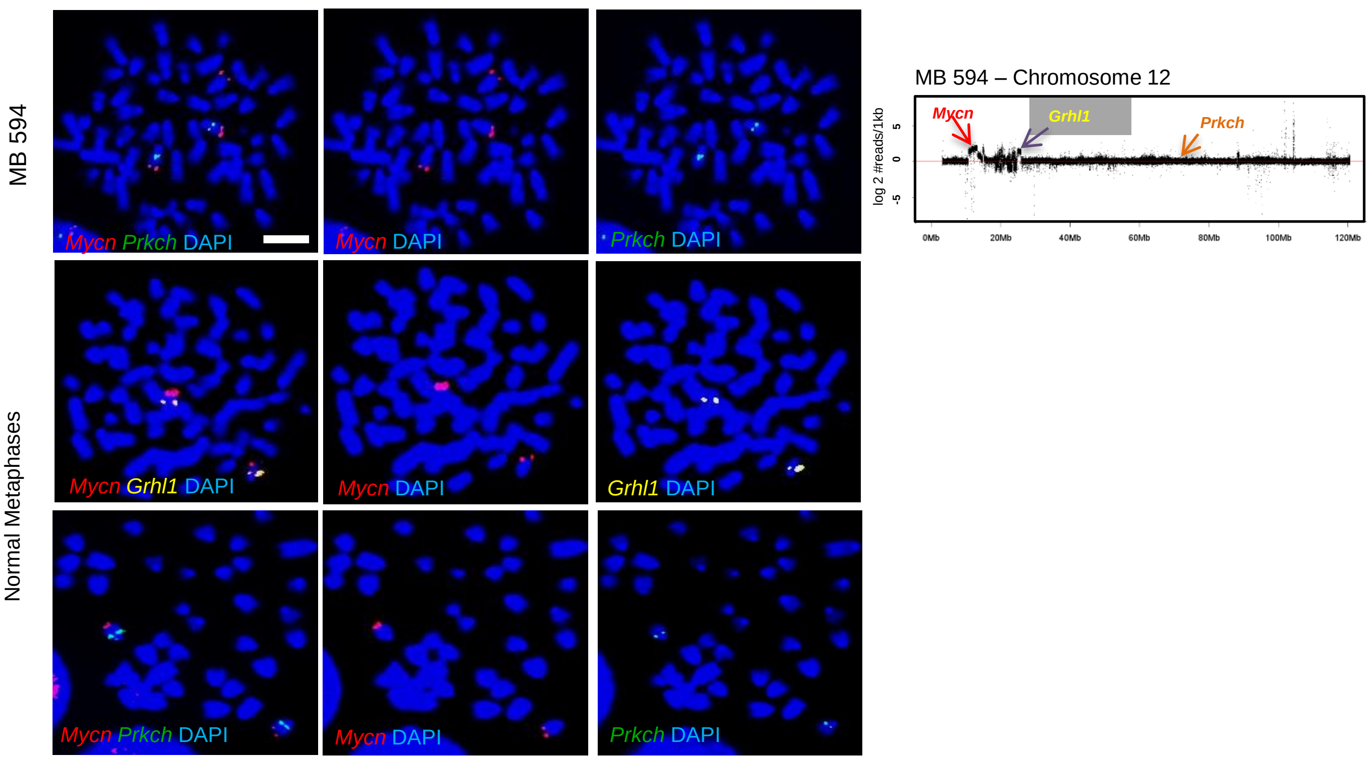

Supplementary Figure 3: **Amplifications of *Myc* and *Mycn* are linked with catastrophic events**

a. Amplifications of *Myc* and *Mycn* may possibly facilitate catastrophic events. FISH analysis with probes for *Myc* (RP23 98D8, green) or *Mycn* (RP23 10C3, red) in combination with probes matching for loci affected by complex genome rearrangements (*Mtbp1*, RP23 288J22 and *Grhl1*, RP23 431C5) showed subpopulations of cells with amplifications of *Myc* or *Mycn* but without gain associated with complex genome rearrangements, suggesting that amplifications of *Myc* or *Mycn* might potentially occur before the catastrophic event. Quantifications of the signals for the control probes *Rpa7a* and *Prkch* are shown in Supplementary Figure 3b and c. Magnification, X1000. **b.** Top panel showing amplifications of *Myc* (RP23 98D8) in comparison to the control probe *Rrp7a* (RP23 25N17) in metaphase spreads from tumor cells. Bottom two panels showing FISH for probes *Myc, Mtbp1* (RP23 288J22) and *Rrp7a* in metaphase spreads from a healthy control mouse. Magnification X1000. **c.** Top panel showing amplification of *Mycn* (RP23 10C3) in comparison to *Prkch* (RP23 440M11) in metaphase spreads from tumor cells. Bottom two panels showing FISH for *Mycn, Grhl1* (RP23 431C5) and *Prkch* in metaphase spreads from a healthy control mouse. Magnification X1000.

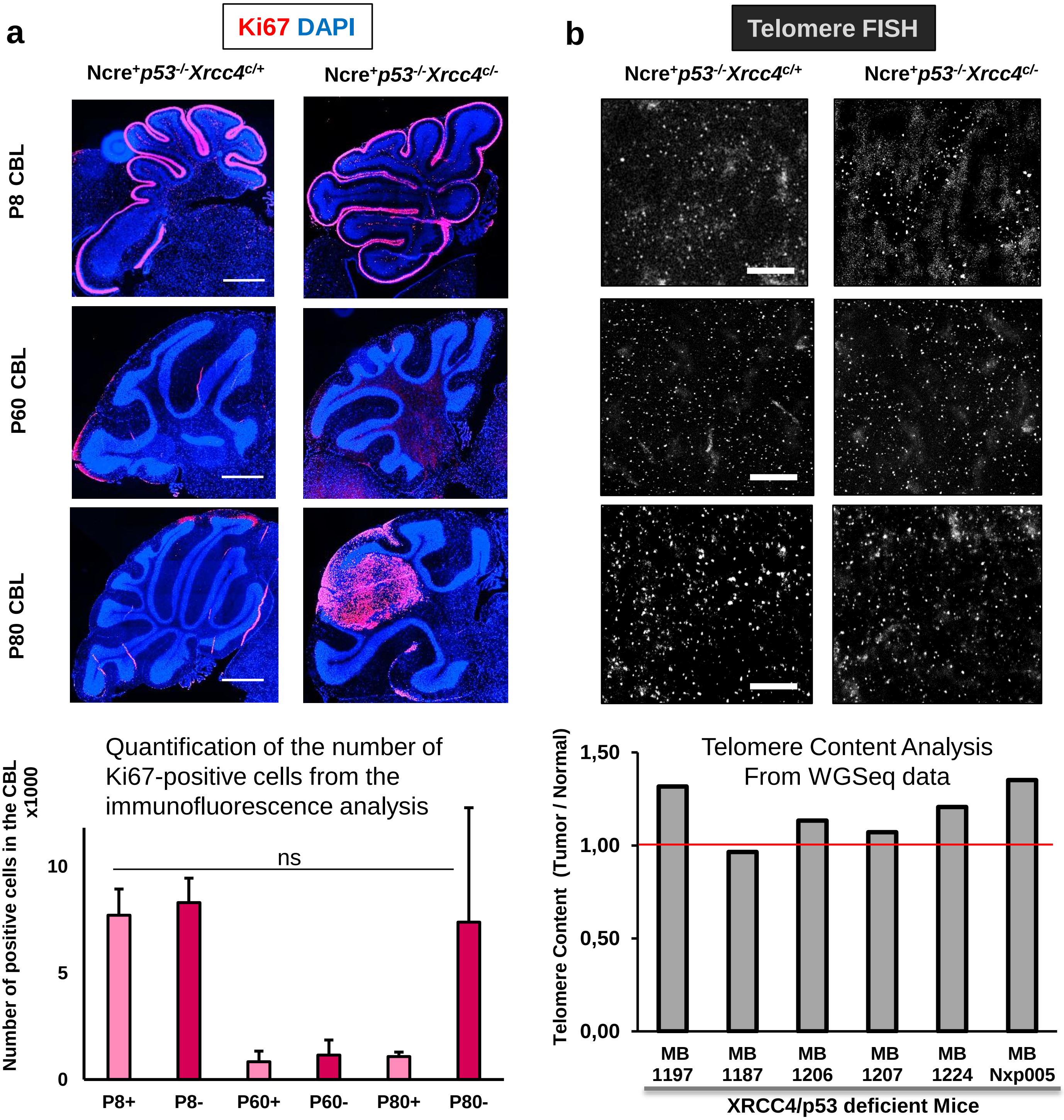

Supplementary Figure 4: Telomere FISH and immunofluorescence analysis for Ki67 in the brain of Xrcc4/p53 deficient and control heterozygous mice

a. Immunofluorescence for Ki67 and Telomere FISH analysis in the cerebellum of XRCC4/p53 deficient and control mice. Ki67 magnification, X100; Unpaired t-tests were used to test for significance. Between 4 and 12 sections were analyzed per animal. XRCC4 deficient mice, 9 animals; XRCC4 proficient mice, 5 animals. CBL, cerebellum. Unpaired t-tests were used to test for significance. Between 4 and 12 sections were analyzed per animal. XRCC4 deficient mice, 9 animals; XRCC4 proficient mice, 5 animals. CBL, cerebellum. **b**. Telomere FISH magnification X1000. Telomere content analysis from the whole-genome sequencing data was done according to Telomere Hunter[3] and did not show any significant difference between tumor and matched germline controls or between cases with or without complex genome rearrangements

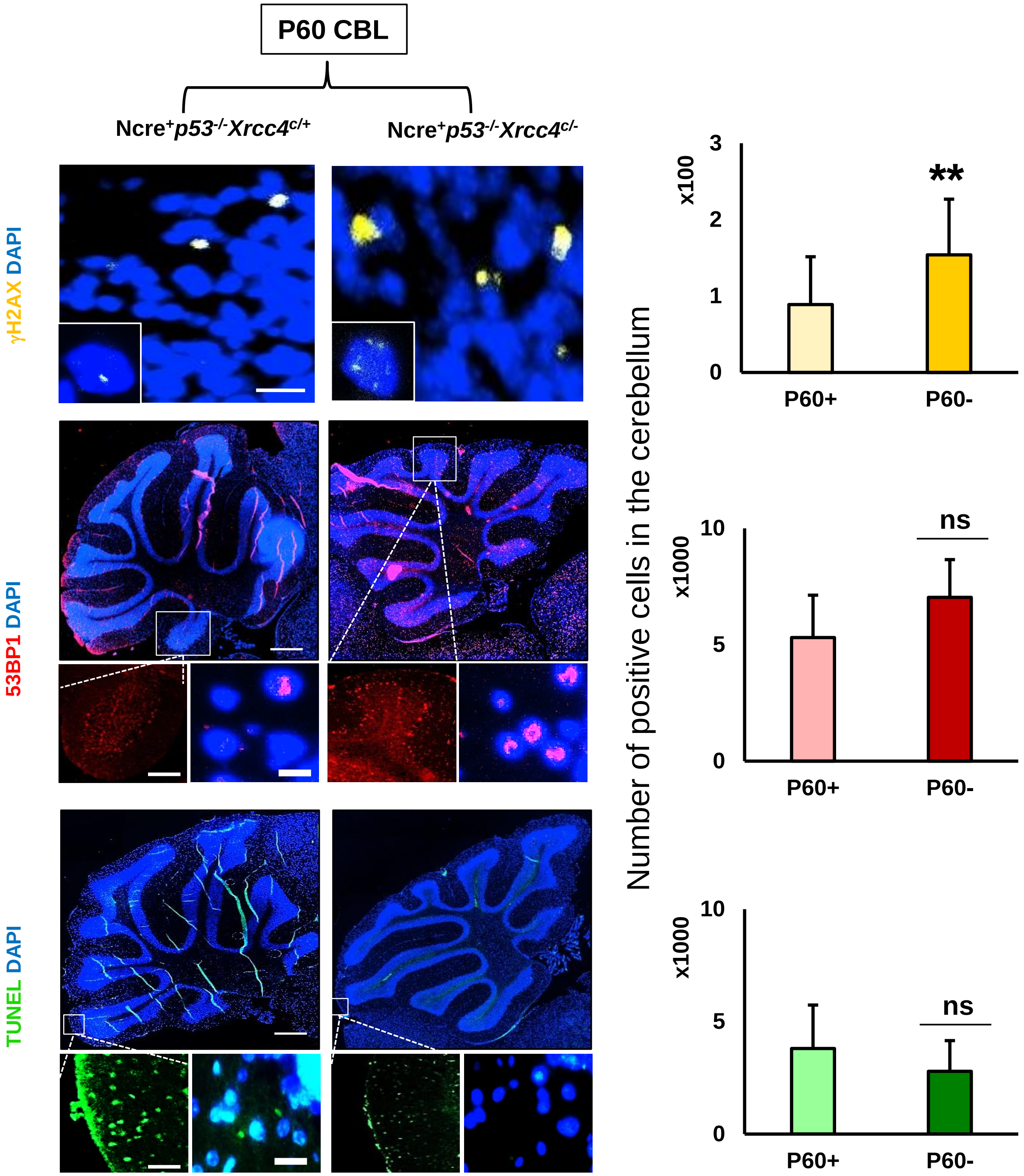

Supplementary Figure 5: Representative images of TUNEL, gH2AX and 53BP1 staining's at intermediate developmental stages with quantifications of the numbers of positive cells

Intermediate time point (P60), yH2AX, 53BP1 and TUNEL staining in the cerebellum of XRCC4/p53 deficient mice and control animals (magnification, X100; insets, X200 and X1000 respectively). Unpaired t-tests were used to test for significance for the quantifications. Between 4 and 12 sections were analyzed per animal. XRCC4 deficient mice, 9 animals; XRCC4 proficient mice, 5 animals.

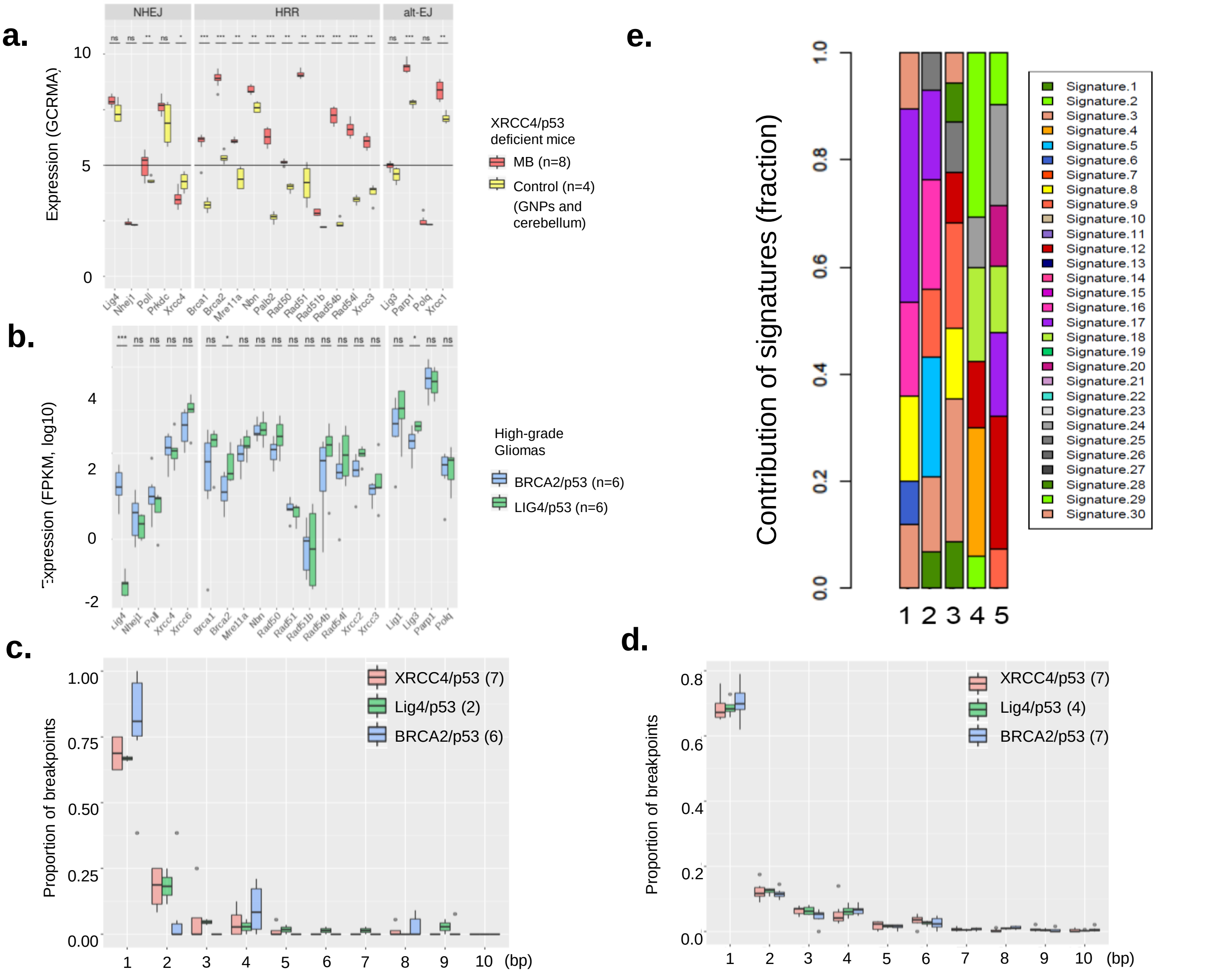

Supplementary Figure 6: **Expression levels for the main repair factors, controls for microhomology analysis (indels) and controls for mutational signature analysis**

Normalized expression levels for repair factors involved in the main DNA DSB repair processes **a.** Red boxes show the expression levels in medulloblastoma cells of XRCC4/p53 mice (n=8) and yellow boxes show the expression levels in the healthy cerebellum (n=2) and in granule neural progenitors (n=2) for age-matched animals of the same genotype (the expression levels for the non-neoplastic cerebellum and for granule neural progenitors were not significantly different and therefore shown as one box). The horizontal line depicts the threshold for which genes are considered to be expressed **b.** Normalized expression levels for repair factors in high grade gliomas developing in BRCA2/p53 (blue boxes) or Lig4/p53 (green boxes) deficient animals. Unpaired t-tests were used to test for significance in a and b. **c**.Size of small deletions related to homology in regions affected by catastrophic events. The plot shows the sizes of the small deletions (<=10bp) within regions affected by catastrophic events. Indels were called by svaba using default threshold and filtering. The differences are not significant (beta-regression analysis). **d.** Size of small deletions related to homology in all regions. The plot shows the sizes of the small deletions (<=10bp) in the whole genome. Indels were called by svaba using default threshold and filtering. The differences are not significant (beta-regression analysis). **e**. Controls for Mutational Signature analysis. As mutational signature 3 was detected in homologous recombination proficient tumors (see figure 4), we wanted to test whether mutational signature 3 might be linked with deficiencies in the p53 pathway, beyond the association with HR defects. Therefore, we did mutational signature analysis for mouse models with published high-coverage WGSeq data. 1 and 2 are murine mammary carcinomas from a STAT1 knock-out model[4] (with a *Rad50* mutation in 1), 3 is a pancreatic tumor from a p53 wild-type/Cdnk2a deficient model[5], 4 and 5 are from a p53 wild-type aflatoxin B induced tumor model[6].

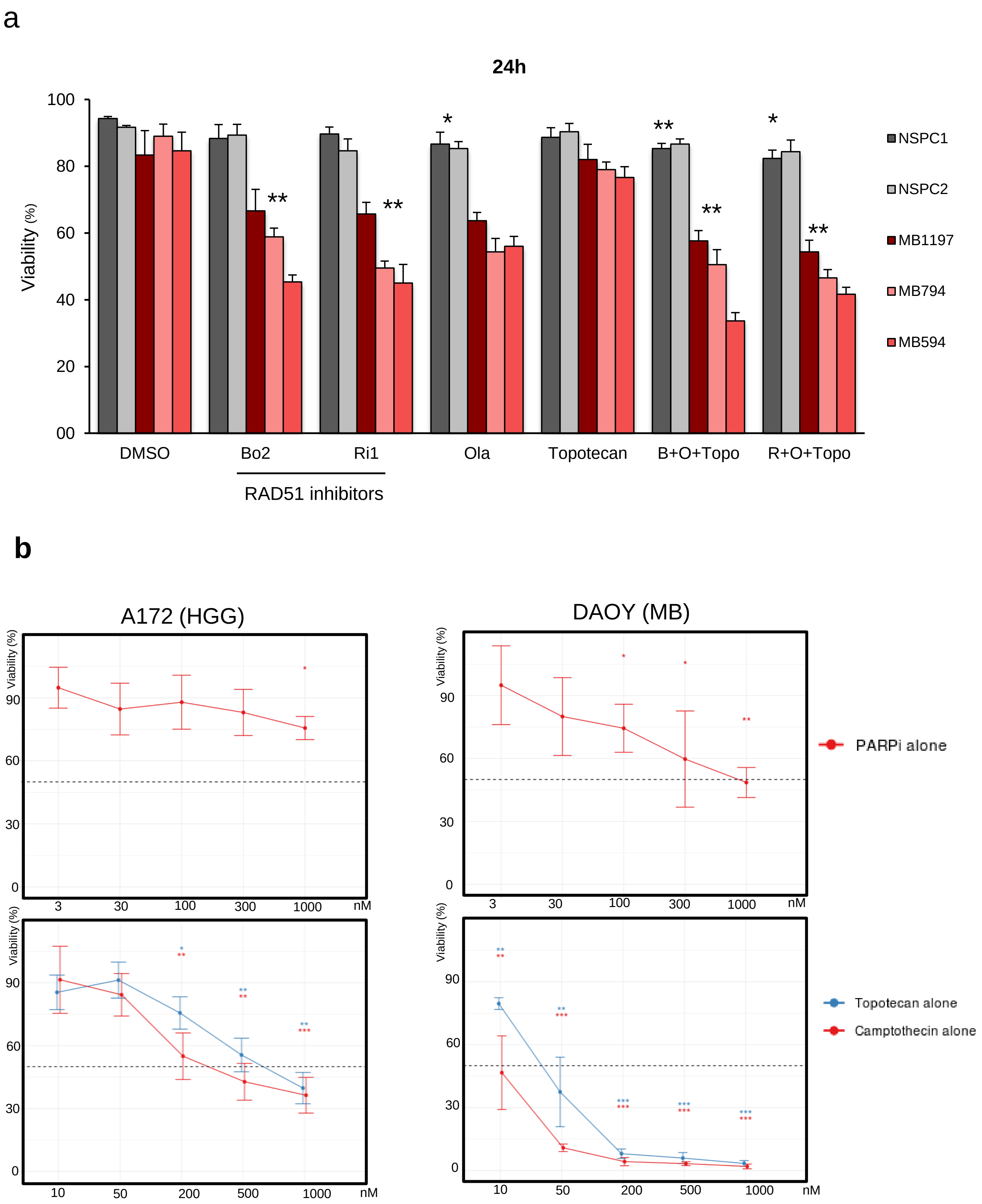

a
24h
Viability (%)
100
80
60
40
20
00
NSPC1
NSPC2
MB1197
MB794
MB594
DMSO
Bo2
Ri1
Ola
Topotecan
B+O+Topo
R+O+Topo
RAD51 inhibitors
b
A172 (HGG)
DAOY (MB)
Viability (%)
90
60
30
0
3
30
100
300
1000
nM
10
50
200
500
1000
PARPi alone
Topotecan alone
Camptothecin alone

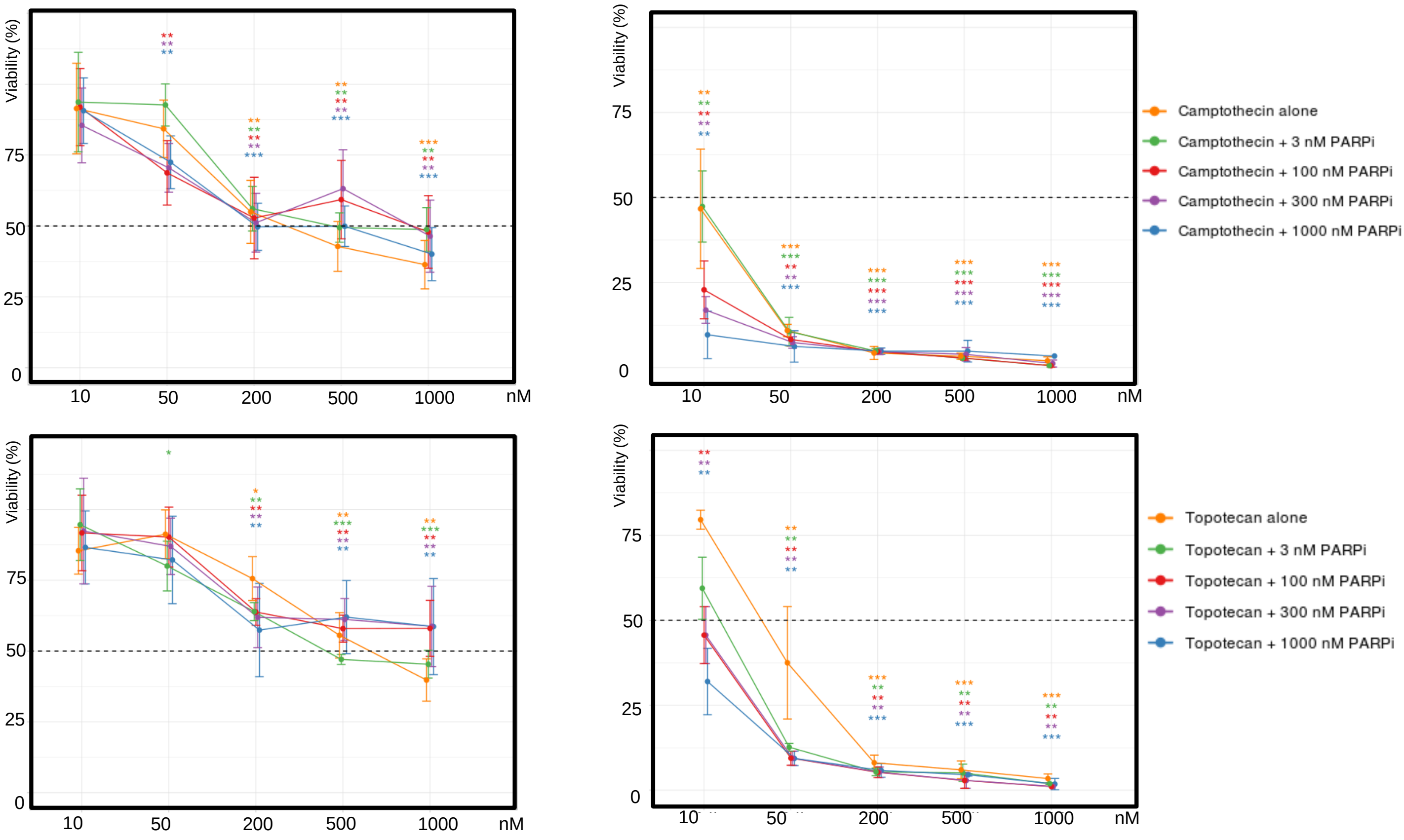

Supplementary Figure 7: **Synthetic lethality in mouse cells, read-out after 24h of treatment. Cytotoxicity of PARPi in combination with topoisomerase inhibitors in human medulloblastoma and high-grade glioma cells with complex genome rearrangements**.

a. Early time point (24h) for synthetic lethality approaches targeting medulloblastoma cells from XRCC4/p53 deficient mice. Medulloblastoma cells (MB594, MB794 and MB1197) from Nestin-Cre *p53* $^{-/-}$ *Xrcc4* $^{c/-}$ and Nestin-Cre *p53* $^{-/-}$ *Xrcc4* $^{c/c}$ mice as well as control neural progenitor cells (NSPC1 and NSPC2) from Nestin-Cre *p53* $^{-/-}$ *Xrcc4* $^{+/-}$ *mice* were treated with RAD51 inhibitors to block HR (B02, 10uM and Ri1, 10uM), PARP inhibitor (Olaparib, 5uM), topotecan to induce DNA damage (100nM) or with combinations (B02 10uM, Olaparib 5uM and Topotecan 100nM or Ri1 10uM, Olaparib 5uM and Topotecan 100nM). Cell viability values are shown as averages +/- standard deviation for 3 independent experiments. ANOVA was used to test for significance. **b.** The effect of single drug treatments and combinations with PARPi (Talazoparib, 3nm-1µM), Topotecan or Camptothecin (10nm-1µM) on human medulloblastoma (DAOY) and glioblastoma (A172) cells with complex genome rearrangements, measured after 72 h. Cell viability values are shown as averages +/- standard deviation for 3 to 4 independent experiments as percentages of the values for solvent-only cells.

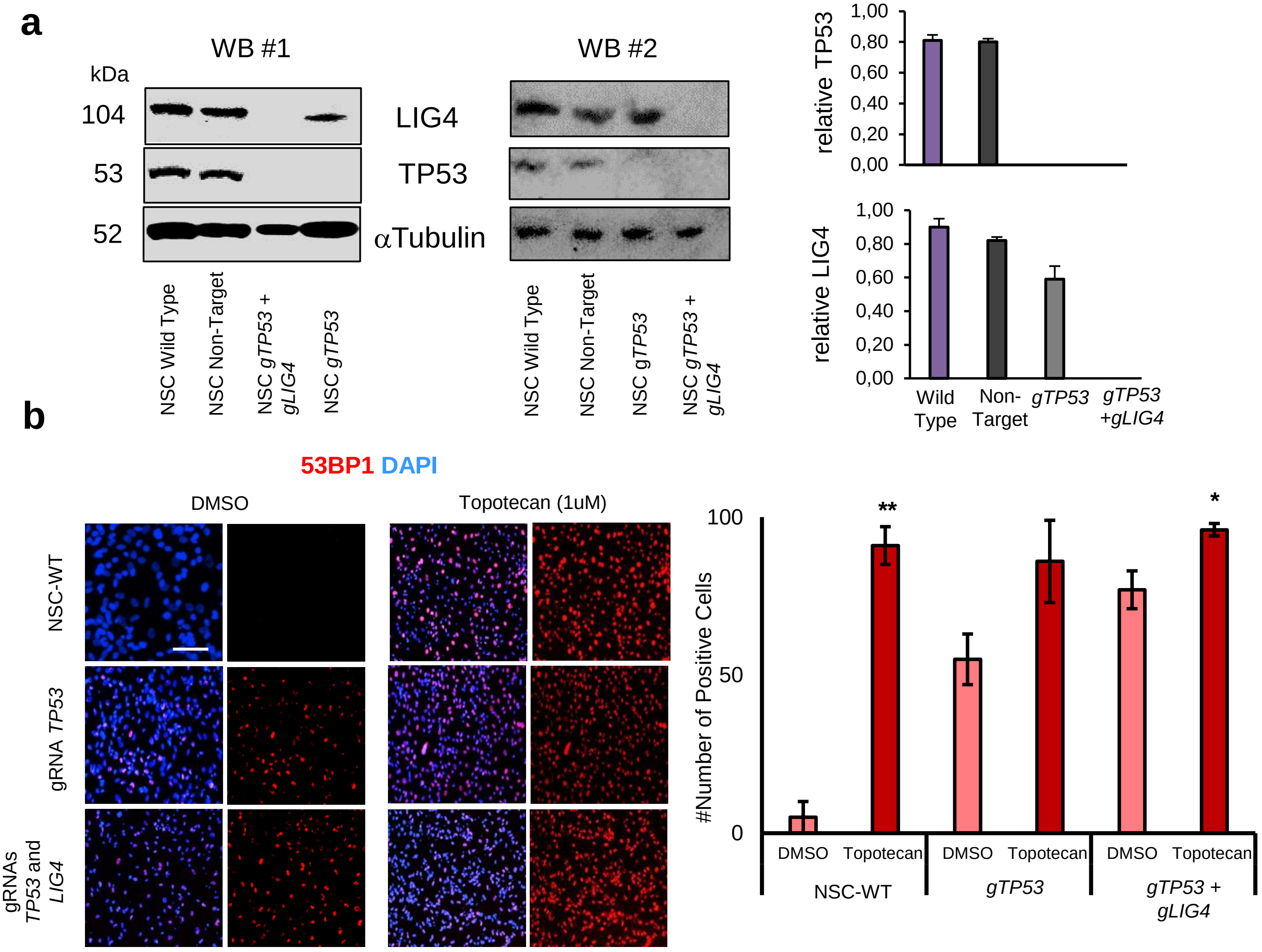

Supplementary Figure 8: Validation of CRIPSR/Cas9 experiments and immunofluorescence for 53BP1

a. Western blot showing whole protein lysates for human neural stem cells (NSCs) for wild type, non target, CRISPR/Cas-mediated disruption of *TP53* and/or *Lig4*. Bar graph analysis on the right shows average relative LIG4 and TP53 in comparison to Alpha-Tubulin from the two western blots (WB) ± SD for two independent experiments. **b.** 53BP1 immunofluorescence for NSC DMSO control and NSC topotecan (1uM) treated cells after 48h. Magnification X100. Bar graph on the right shows the number of positive cells for 53BP1 from a total of 100 counted cells. Error bars indicate ±SD for three independent experiments

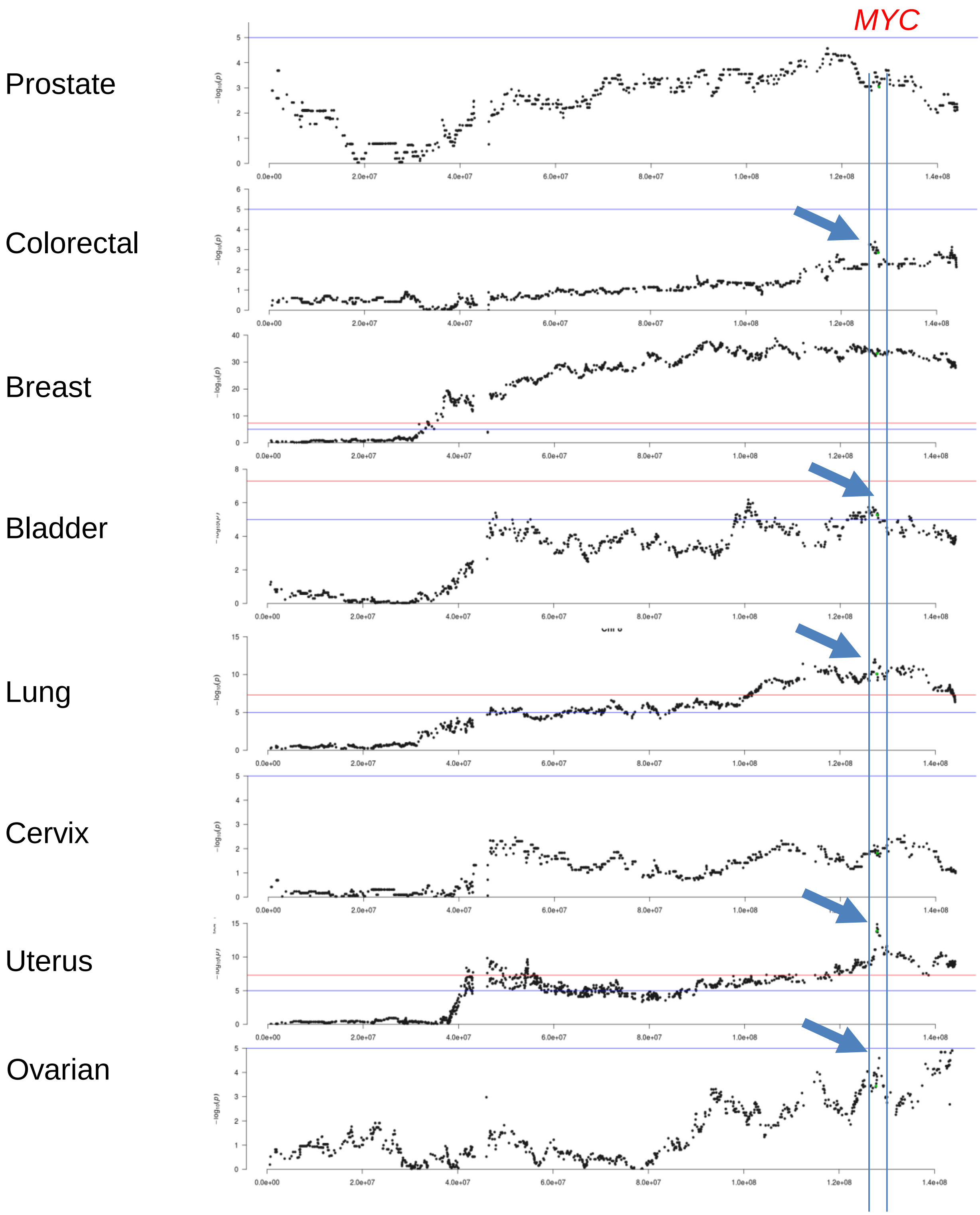

Supplementary Figure 9: **Pan cancer analysis of the association between chromothripsis and MYC status in human tumors**

Pan-cancer analysis of regions with copy-number gains significantly associated with chromothripsis. *MYC* gains are strongly linked with chromothripsis across multiple tumor entities (re-analysis of TCGA data). Y axis, -log10 (p value) for the association; x axis, genomic position.

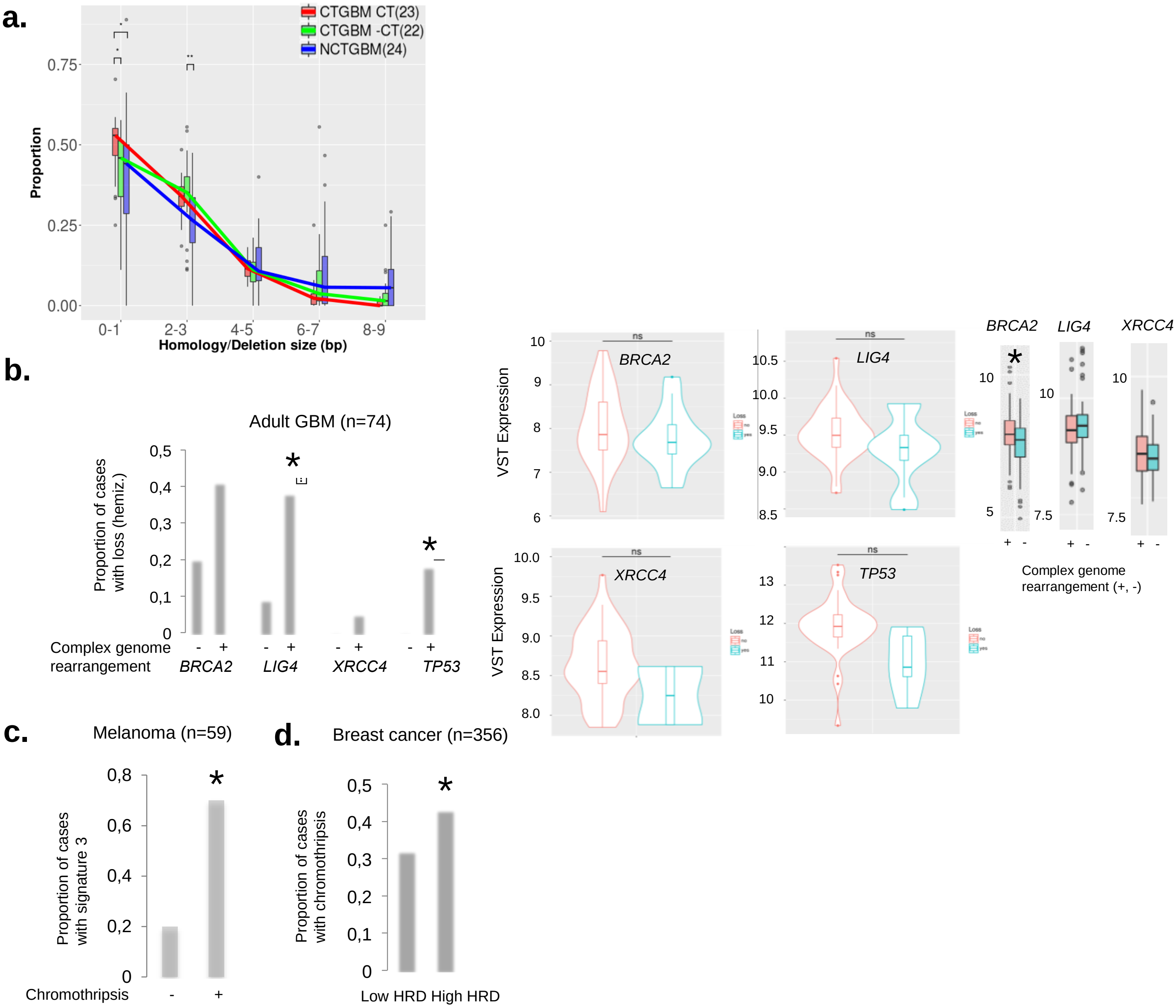

Supplementary Figure 10: **Microhomology at the breakpoints in human GBM and Pan cancer analysis of the association between repair deficiencies and chromothripsis**

a. Size of the microhomology utilized by the structural variants junctions in different regions in human GBM. “CTGBM CT” indicates breakpoints within chromothriptic regions, “CTGBM -CT” indicates breakpoints outside chromothriptic regions for tumors showing chromothripsis, and “NCTGBM” indicates all breakpoints in tumors without chromothripsis. Beta-regression was used to test for significance. **b.** Association between deficiency in repair processes and chromothripsis in adult glioblastoma. Link between loss of repair factors and chromothripsis in adult GBM cases (reanalysis of TCGA data). Left panel, proportion of cases with loss of the genomic loci where *BRCA2*, *LIG4*, *XRCC4* and *TP53* are located, respectively, among cases with or without chromothripsis. Right panels, expression levels for *BRCA2*, *LIG4*, *XRCC4* and *TP53* in cases with (blue) or without (red) hemizygous loss of the respective genomic loci (violine plots) and in cases with or without chromothripsis (box plots, far right). Unpaired t-tests were used to test for significance. **c.** Link between chromothripsis and mutational signature 3 in melanoma. Unpaired t-tests were used to test for significance**. d.** Link between chromothripsis and Homologous Recombination Deficiency index in breast cancer (reanalysis of data from Nik-Zainal et al, Nature 2016). Low HRD, up to 5; High HRD, 6 and more. Unpaired t-tests were used to test for significance.

Supplementary References

1. Poschl J, Stark S, et.al., Acta Neuropathol 2014 Jul;128(1):123-36. doi: 10.1007/s00401-014-1297-8. Genomic and transcriptomic analyses match medulloblastoma mouse models to their human counterparts.

2. Akgül S, Li Y, et al., Cell Reports 2018 Jul 10;24(2):463-478.e5. doi: 10.1016/j.celrep.2018.06.050. Opposing Tumor-Promoting and -Suppressive Functions of Rictor/mTORC2 Signaling in Adult Glioma and Pediatric SHH Medulloblastoma.

3. Genomic footprints of activated telomere maintenance mechanisms in cancer. Sieverling et al, Nature Communications, accepted for publication

4. Griffith et al, Cell Rep. 2016 Sep 27;17(1):249-260. doi: 10.1016/j.celrep.2016.08.076. Truncating Prolactin Receptor Mutations Promote Tumor Growth in Murine Estrogen Receptor-Alpha Mammary Carcinomas.

5. Mueller et al, Nature. 2018 Feb 1;554(7690):62-68. doi: 10.1038/nature25459. Epub 2018 Jan 24. Evolutionary routes and KRAS dosage define pancreatic cancer phenotypes.

6. Mi Ni Huang et al, Genome Res. 2017 Sep; 27(9): 1475–1486. Genome-scale mutational signatures of aflatoxin in cells, mice, and human tumors
