# Supplementary material for: Defective DNA damage repair leads to frequent catastrophic genomic events in murine and human tumors
Source: Nat Commun. 2018 Nov 12;9:4760. doi: 10.1038/s41467-018-06925-4 (PMC6232171; doi:10.1038/s41467-018-06925-4)
Supplement: Supplementary file 3 — Description of Additional Supplementary Files [file 41467_2018_6925_MOESM3_ESM.docx]

**Description of Additional Supplementary Files**

File Name: Supplementary Data 1

Description: Summary of the mouse tumors analyzed in this study, including scoring for catastrophic events and status of Myc and Mycn.

File Name: Supplementary Data 2

Description: Scoring for complex genome rearrangements based on the Shatterseek algorithm combined with manual curation by visual inspection to remove false positive events detected by the program.

File Name: Supplementary Data 3

Description: Link between chromothripsis and gains of MYC or MYCN in human SHH MB and GBM.

File Name: Supplementary Data 4

Description: Scoring for complex genome rearrangements in human MBs and HGGs for which pathogenic mutations in DNA repair genes were detected
