# Supplementary figures and images for: Author Correction: Defective DNA damage repair leads to frequent catastrophic genomic events in murine and human tumors
Source: Nat Commun. 2026 Apr 20;17:3611. doi: 10.1038/s41467-026-71976-x (PMC13096407; doi:10.1038/s41467-026-71976-x)

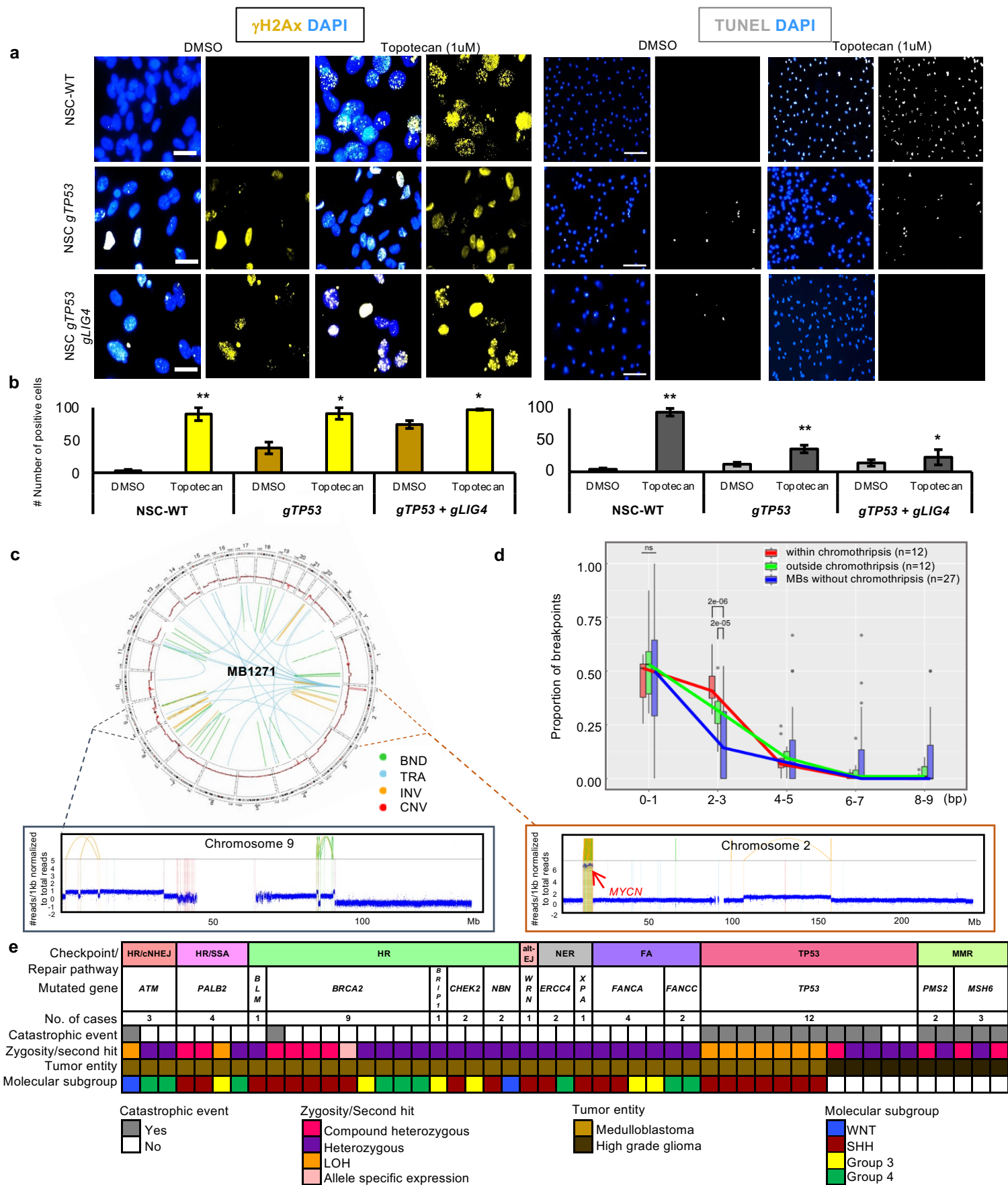

Supplement: Supplementary file 1 — Corrected Fig. 5 [file 41467_2026_71976_MOESM1_ESM.pdf]
